# Challenges in rural maternal health: how received public services and policy awareness affect health knowledge and practices

**DOI:** 10.3389/fpubh.2024.1514522

**Published:** 2025-01-14

**Authors:** Jie Yang, Jun Chen, Yuyang Xie, Yunjie Liu, Junhao Wu, Yangyuan Li, Jingchun Nie

**Affiliations:** Center for Experimental Economics in Education, Faculty of Education, Shaanxi Normal University, Xi’an, China

**Keywords:** received public services, policy awareness, health knowledge, health practices, pregnancy, rural areas

## Abstract

**Purpose:**

This study evaluates the effectiveness of rural maternal health services in improving pregnant women’s health knowledge, practices, and outcomes in northwestern China, focusing on the roles of received public services and policy awareness.

**Methods:**

Baseline surveys were conducted in rural Shaanxi Province in 2021 and 2023, involving 1,152 pregnant women from 85 townships, selected via multistage cluster random sampling. Data were collected through structured face-to-face interviews, covering health knowledge and behaviors. Statistical analyses were performed to assess the impact of maternal health services.

**Results:**

Both received public services (*Coefficient*: 0.130, 95% *CI*: 0.015–0.246) and policy awareness (*Coefficient*: 0.114, 95% *CI*: 0.001–0.227) significantly improved nutrition and health knowledge but had limited impact on prenatal checkups or health outcomes. Policy awareness (*OR*: 3.826, 95% *CI*: 2.743–5.337) significantly increased picking up free folic acid, however, the rate of taking folic acid remained low.

**Conclusion:**

While received public services and policy awareness improved nutrition and health knowledge, and policy awareness increased picking up free folic acid, they did not significantly influence prenatal checkups or health outcomes. More targeted efforts are needed to foster consistent health practices and improve maternal health outcomes in rural areas.

## 1 Introduction

Maternal health is widely recognized as a cornerstone of infant nutrition, health, and development (1, 2). Ensuring the well-being of pregnant women has profound implications not only for birth outcomes but also for the long-term health of the next generation. The Developmental Origins of Health and Disease (DOHaD) theory posits that a mother’s health during pregnancy can have lasting effects on a child’s future health, influencing risks for conditions such as obesity, cardiovascular disease, and diabetes (3, 4). For example, folic acid deficiency during early pregnancy has been proven to be associated with neural tube defects in newborns (5, 6), while anemia during pregnancy has been extensively associated with adverse outcomes, including low birth weight, preterm birth, and maternal mortality (7–9).

Public health interventions, particularly those targeting maternal health, are vital in reducing these risks. Iron and folic acid supplementation (IFAS), along with regular prenatal checkups, has been shown to significantly improve maternal and infant health outcomes (10–13). These services can mitigate complications and enhance birth outcomes by addressing nutritional deficiencies and monitoring maternal health throughout pregnancy (14–17). In response, many countries have implemented policies to enhance maternal health, including mandatory food fortification and free supplementation programs (18). Brazil, Mexico, and Canada, for instance, have enforced food fortification policies to increase iron intake (19), while Kenya provides free iron and folic acid supplements to pregnant women through its IFAS program (20). India’s National Rural Health Mission offers free prenatal checkups and educates pregnant women through community health workers (21).

China has also taken significant steps to improve maternal and child health. In 2009, the government started to distribute free folic acid supplements to women of childbearing age nationwide (22). In 2010, China launched the National Free Preconception Health Examination Program, ensuring that all pregnant women have access to essential prenatal care services (23). Currently, pregnant women are eligible to receive five free prenatal checkups at designated public hospitals, community health centers, or township clinics. However, despite these efforts, significant challenges remain in improving maternal health knowledge, behaviors, and outcomes—particularly in rural areas. Research indicates that antenatal care visits are significantly more common in urban areas, with a rate of 76–77%, compared to only 46% in rural areas, highlighting a substantial disparity in the utilization of maternal and child health services (24, 25).

In rural China, health services and folic acid supplements are underutilized despite their availability. For example, only 44.5% of participants in one study took free folic acid supplements before and during pregnancy, even though 76.4% received the supplements free of charge (26, 27). Similar studies (28, 29) highlighted persistent maternal health challenges, including higher rates of anemia (43–46% in rural areas vs. 30–35% in urban areas), malnutrition, and postpartum depression (13% prevalence). These findings suggest that improving maternal health outcomes requires more than service provision––it also demands addressing gaps in health knowledge and behaviors.

Exploring pregnant women’s health knowledge and behaviors is therefore essential. While previous studies have examined maternal nutrition and health knowledge, health behaviors, and overall health outcomes, they often focus on demographic and socioeconomic factors (30–33), leaving other influences, such as the effectiveness of public health management practices, underexplored. Several studies suggest that maternal and child health services can improve pregnant women’s knowledge of nutrition and health (34–36), but few have specifically analyzed how these services function in rural China.

This study aims to address this research gap by examining the health knowledge, behaviors, and outcomes of pregnant women in rural northwest China, with a particular focus on how maternal health services impact these outcomes. We first describe the women’s nutritional health knowledge, health behaviors (including prenatal checkups, picking up free folic acid, and taking folic acid), and health outcomes. Then, we analyze whether and how received public services and policy awareness influence these aspects, ultimately evaluating the effectiveness of maternal health services in rural China. By identifying key factors that affect maternal health in rural regions, this study provides valuable insights for policymakers seeking to improve health outcomes for pregnant women in these areas.

## 2 Materials and methods

### 2.1 Study population and sampling

The data used in this study were drawn from baseline surveys on maternal and infant nutrition and health, conducted by Shaanxi Normal University in rural areas of Shaanxi Province in 2021 and 2023. The project was carried out in five cities across central and southern Shaanxi, with two counties randomly selected from each city, totaling ten counties. Surveys were conducted among rural pregnant women in March 2021, December 2021, and March 2023.

We employed a multistage cluster random sampling method to identify potential participants. The sampling process involved three main steps: first, selecting 10 economically disadvantaged counties from 5 prefecture-level cities in central and southern Shaanxi Province. In each county, the township where the county government is located was excluded due to its higher economic and urbanization levels. Townships were either fully included or randomly sampled if exceeding 10. For ethical and practical reasons, and to ensure the participants were representative of the target population, the inclusion criteria for our participants were as follows: (1) aged between 28 and 40 years; (2) had resided in the locality for at least 2 years; (3) had no history of mental illness or other severe health conditions; (4) priority was given to pregnant women with younger gestational ages. After completing the four sampling steps and excluding cases with missing key variables, the final valid sample for analysis consisted of 1,152 pregnant women from 85 townships in 10 counties. This included 592 cases from March 2021, 228 cases from December 2021, and 332 cases from March 2023.

### 2.2 Survey method

In this study, a questionnaire survey method was employed, with trained interviewers conducting face-to-face interviews with each participant. The questionnaire included information on the pregnant women’s sociodemographic characteristics, health status, prenatal checkups, and folic acid intake. Prior to the interviews, informed consent forms were distributed to eligible participants, detailing the project’s objectives, procedures, potential risks, benefits, and privacy protection. To ensure accuracy and consistency during data collection, interviewers underwent centralized training, and a pre-survey was conducted with 20 participants before the large-scale data collection. All interview questions were displayed on tablets, with interviewers asking questions one by one while recording the answers. Each interview was conducted privately between the interviewer and the pregnant woman to prevent interference from other family members.

### 2.3 Definition and measurement of variables

The dependent variables in this study involve three aspects of pregnant women’s health: health knowledge, practices, and outcomes. Specifically, these include nutrition and health knowledge, prenatal checkups, picking up free folic acid, taking of folic acid, and health outcomes. The nutrition and health knowledge of pregnant women was assessed using a questionnaire consisting of 24 items, with 12 questions addressing maternal nutrition and health, and 12 focusing on neonatal feeding. Each item provides 4–5 answer choices, including one correct answer, one “Do not know” option, and others serving as distractors. One correct answer is awarded one point, yielding a maximum score of 24. Consequently, the nutrition and health knowledge score is treated as a continuous variable. For statistical analyses, the knowledge scores were standardized to ensure comparability across models. Prenatal checkups is defined according to the frequency recommended by the “Guidelines for Preconception and Prenatal Care (2018),” which suggests 7 to 11 checkups during pregnancy, with additional visits for high-risk pregnancies. It is calculated based on participants’ self-reported number of prenatal checkups. If the number of checkups reported meets or exceeds the recommended frequency, the variable is assigned a value of 1; otherwise, it is assigned a value of 0. The variables picking up free folic and taking folic acid are defined based on two distinct survey questions. For picking up free folic, the question is, “Have you picked up free folic acid?” For taking folic acid, the question is, “Have you taken folic acid before or during this pregnancy?” Respondents who answered “Yes” are assigned a value of 1, while those who answered “No” are assigned a value of 0. The variable health outcomes is constructed based on six recent symptoms from the questionnaire and anemia status. Recent symptoms include fever, sore throat, cough, diarrhea, stomach pain, asthma, headache or dizziness, and joint or muscle pain, with each “Yes” response scored as 1 and “No” as 0. Anemia status, determined by hemoglobin levels (37), is scored as 1 for anemia and 0 for non-anemia. These variables were summed to generate the variable health outcomes (range: 0–7), with higher scores indicating poorer health outcomes.

The independent variables in this study include two sub-dimensions of maternal health services: received public services and policy awareness. Received public services and policy awareness are defined based on two distinct survey questions. For received public services, the question is, “During your pregnancy and early childhood of your baby (e.g., vaccinations, check-ups), has anyone provided management services for you, such as notifications, reminders, or consultations?” For policy awareness, the question is, “Were you ever informed that you could receive free folic acid supplements and advised to attend prenatal checkups?” Respondents who answered “Yes” are assigned a value of 1, while those who answered “No” are assigned a value of 0.

The control variables in this study encompass characteristics of pregnant women at three levels: individual, household, and social. At the individual level, the characteristics include age, trimesters, body mass index (BMI; in weight (kg)/height (m)^2^), completed compulsory education, first pregnancy, and employed. Trimesters is treated as a categorical variable, with values assigned as follows: 0 for the first trimester, 1 for the second trimester, and 2 for the third trimester. BMI is calculated by dividing weight (in kilograms) by the square of height (in meters), measured using standardized scales and measuring tapes under the guidance of local nurses. According to the weight classification criteria for adults outlined in the health industry standards of the People’s Republic of China, BMI is categorized into four groups: < 18.5 kg/m^2^, 18.5 kg/m^2^ ≤ BMI < 24.0 kg/m^2^, 24.0 kg/m^2^ ≤ BMI < 28.0 kg/m^2^, and ≥ 28.0 kg/m^2^. At the household level, we consider whether the pregnant woman resides with her husband and whether the family’s assets fall within the lowest 25% of the sample. The variable family assets in the lowest 25% is defined using an asset index constructed through Principal Component Analysis (PCA) (38), based on responses to nine binary questions about key household assets. Households with an asset index in the bottom quartile are assigned a value of 1, and all others are assigned 0. At the social level, factors include the distance between the pregnant woman’s primary residence and the hospital where she is registered, as well as the survey round. Survey round is treated as a categorical variable indicating the timing of surveys among rural pregnant women. Data were collected during three phases: 0 = March 2021, 1 = December 2021, and 2 = March 2023, with each phase involving different participants.

### 2.4 Statistical analysis

The data processing and statistical analyses in this study were performed using STATA 18.0 software. Categorical variables were summarized with frequency and percentage, while continuous variables were presented by the mean and standard deviation (SD) in descriptive analyses. Multiple linear regression analyses and multivariate logistic regression analyses were performed to test the relationship between maternal health services and dependent variables, including nutrition and health knowledge, health practices, and health outcomes. The statistical level of significance was *p* < 0.05 and the outcomes of regression analyses were presented as coefficients or odds ratios (ORs) with their 95% confidence intervals (CIs).

## 3 Results

### 3.1 Characteristics of study population

Table 1 presents the basic demographic characteristics of the 1,152 pregnant women included in this study. The average age of the respondents was 29.0 years (SD = 4.4). Over half of these women had not completed high compulsory education, and the majority (90.3%) did not have stable employment during their pregnancies. Additionally, 65.3% of the pregnant women were experiencing their first pregnancy, and 66.8% were in the second trimester.

**Table 1:** Sociodemographic characteristics of study population.

Variables	N / Mean	(%) / SD
Individual level		
Age	29.0	4.4
Trimesters		
First trimester	111	9.6
Second trimester	770	66.8
Third trimester	271	23.5
Body mass index (kg/m^2^)		
BMI < 18.5	16	1.4
18.5 ≤ BMI < 24	402	34.9
24 ≤ BMI < 28	467	40.5
BMI ≥28	263	22.8
Missing	4	0.4
Completed compulsory education		
Yes	500	43.4
No	652	56.6
First pregnancy		
Yes	752	65.3
No	400	34.7
Employed		
Yes	112	9.7
No	1,040	90.3
Household level		
Living with husband		
Yes	772	67.0
No	380	33.0
Family asset in lowest 25%		
Yes	292	25.3
No	860	74.7
Social level		
Distance to hospital	2.5	1.1
Survey round		
Survey_Mar2021	592	51.4
Survey_Dec2021	228	19.8
Survey_Mar2023	332	28.8

### 3.2 Descriptive statistics of maternal health services and health knowledge, practices, and outcomes

In Table 2, we describe the status of maternal health services and pregnant women’s health knowledge, practices, and outcomes. Among the 1,152 pregnant women, 692 (60.1%) received public services during their pregnancies; however, 58.0% of the respondents remained unaware of maternal health services policies, including the provision of free folic acid and the promotion of prenatal checkups.

**Table 2:** Descriptive statistics of maternal health services and health knowledge, practices, and outcomes.

Variables	N / Mean	(%) / SD
Panel A: maternal health services		
Received public services	692	(60.1)
Policy awareness	460	(42.0)
Panel B: health knowledge, practices, and outcomes		
Nutrition and health knowledge	11.3	3.5
Prenatal checkups	846	(73.4)
Picking up free folic acid	864	(75.0)
Taking folic acid	512	(44.4)
Health outcomes	1.1	1.1

In terms of nutrition and health knowledge, the mean score was 11.3 (SD = 3.5). Among all 1,152 women, 864 (75.0%) reported having picked up free folic acid before or during pregnancy, and only 512 (44.4%) reported taking folic acid before or during this pregnancy. 73.4% of pregnant women underwent prenatal checkups. The mean health outcomes score of 1.1 indicated that, on average, pregnant women experienced just over one recent symptom or health issue from the assessed range of health problems.

### 3.3 The impact of maternal health services on pregnant women’s nutrition and health knowledge

The impact of maternal health services on pregnant women’s nutrition and health knowledge is illustrated in Table 3. Results show that both received public services (*Coefficient*: 0.130, 95% *CI*: 0.015–0.246) and policy awareness (*Coefficient*: 0.114, 95% *CI*: 0.001–0.227) were significantly associated with a positive impact on nutrition and health knowledge. Additionally, a higher level of nutrition and health knowledge was observed in expectant women with advanced educational qualifications, more affluent socioeconomic status, and steady employment, as well as those expecting their first child.

**Table 3:** Results of multiple linear regression analyses of variables related to nutrition and health knowledge.

	Nutrition and health knowledge (standardized score)	
	Coefficient (95% CI)	*p*-value
Maternal health services		
Received public services	0.130 (0.015–0.246)	0.027
Policy awareness	0.114 (0.001–0.227)	0.048
Characteristics of pregnant women		
Age	0.002 (−0.011–0.015)	0.762
Trimesters (0 = first trimester 1 = second trimester 2 = third trimester)	0.111 (0.014–0.208)	0.026
BMI (BMI < 18.5 as reference)		
18.5 ≤ BMI < 25	−0.154 (−0.611–0.302)	0.507
25 ≤ BMI < 30	−0.164 (−0.622–0.293)	0.481
BMI ≥ 30	−0.229 (−0.692–0.234)	0.332
Completed compulsory education (1 = yes)	0.639 (0.523–0.755)	<0.001
First pregnancy (1 = yes)	0.178 (0.052–0.304)	0.006
Employed (1 = yes)	0.383 (0.197–0.570)	<0.001
Living with husband (1 = yes)	−0.057 (−0.171–0.056)	0.321
Family asset in lowest 25% (1 = yes)	−0.316 (−0.441–-0.192)	<0.001
Distance to hospital	−0.007 (−0.055–0.041)	0.763
Survey round (0 = survey_Mar2021 1 = survey_Dec2021 2 = survey_Mar2023)	−0.018 (−0.080–0.044)	0.572

Survey round is categorical variable indicating the timing of surveys conducted among rural pregnant women. Data were collected during three phases: 0 = March 2021, 1 = December 2021, and 2 = March 2023, with each phase involving different participants. Nutrition and health knowledge scores were standardized for statistical analyses. Coefficients are estimated using multiple linear regression, with 95% confidence intervals (CI) reported.

### 3.4 The impact of maternal health services on pregnant women’s health practices

Logistic regression analyses in Table 4 show that expectant women who were aware of the maternal health services policies had 3.8 times higher odds of picking up free folic acid (*OR*: 3.826, 95% *CI*: 2.743–5.337) than their counterparts and were more likely to take folic acid before or during this pregnancy (*OR*: 1.822, 95% *CI*: 1.409–2.356). Received public services showed no significant association with pregnant women’s health practices.

**Table 4:** Results of multivariate logistic regression analyses of variables related to health practices.

	Prenatal checkups		Picking up free folic acid		Taking folic acid	
	OR (95% CI)	*p*-value	OR (95% CI)	*p*-value	OR (95% CI)	*p*-value
Maternal health services						
Received public services	0.813 (0.598–1.107)	0.189	0.958 (0.712–1.289)	0.775	1.101 (0.844–1.437)	0.479
Policy awareness	1.192 (0.884–1.608)	0.249	3.826 (2.743–5.337)	<0.001	1.822 (1.409–2.356)	<0.001
Characteristics of pregnant women						
Age	0.989 (0.955–1.025)	0.552	0.992 (0.958–1.027)	0.641	1.058 (1.026–1.091)	<0.001
Trimesters (0 = first trimester 1 = second trimester 2 = third trimester)	0.289 (0.220–0.379)	<0.001	0.787 (0.608–1.017)	0.067	0.977 (0.782–1.222)	0.840
BMI (BMI < 18.5 as reference)						
18.5 ≤ BMI < 25	0.641 (0.138–2.972)	0.570	0.812 (0.217–3.041)	0.757	2.526 (0.782–8.164)	0.122
25 ≤ BMI < 30	0.693 (0.149–3.218)	0.639	0.656 (0.175–2.456)	0.531	2.110 (0.652–6.835)	0.213
BMI ≥ 30	0.769 (0.163–3.617)	0.739	0.778 (0.204–2.965)	0.713	2.365 (0.722–7.753)	0.155
Completed compulsory education (1 = yes)	1.012 (0.743–1.378)	0.940	0.867 (0.639–1.178)	0.362	1.084 (0.831–1.414)	0.554
First pregnancy (1 = yes)	0.728 (0.519–1.022)	0.066	1.009 (0.723–1.408)	0.958	0.425 (0.318–0.569)	<0.001
Employed (1 = yes)	0.786 (0.484–1.278)	0.332	1.152 (0.686–1.937)	0.592	1.244 (0.812–1.906)	0.316
Living with husband (1 = yes)	0.758 (0.559–1.028)	0.075	1.030 (0.765–1.386)	0.847	1.227 (0.945–1.593)	0.124
Family asset in lowest 25% (1 = yes)	0.708 (0.515–0.972)	0.033	0.745 (0.542–1.024)	0.070	0.786 (0.589–1.048)	0.101
Distance to hospital	0.870 (0.764–0.992)	0.038	0.882 (0.774–1.005)	0.060	0.995 (0.891–1.112)	0.931
Survey round (0 = survey_Mar2021 1 = survey_Dec2021 2 = survey_Mar2023)	1.245 (1.054–1.469)	0.010	0.920 (0.782–1.083)	0.316	1.010 (0.876–1.164)	0.892

Survey round is categorical variable indicating the timing of surveys conducted among rural pregnant women. Data were collected during three phases: 0 = March 2021, 1 = December 2021, and 2 = March 2023, with each phase involving different participants. The odds ratios (ORs) are estimated using multivariate logistic regressions, with 95% confidence intervals (CI) reported.

### 3.5 The impact of maternal health services on pregnant women’s health outcomes

From the multiple linear regression analyses in Table 5, the results indicate that neither received public services nor policy awareness was significantly associated with improvements in the health outcomes of pregnant women. Most other variables were also not significantly associated with pregnant women’s health outcomes.

**Table 5:** Results of multiple linear regression analyses of variables related to health outcomes.

	Health outcomes	
	Coefficient (95% CI)	*P*-value
Maternal health services		
Received public services	−0.091 (−0.230–0.049)	0.203
Policy awareness	−0.064 (−0.201–0.072)	0.355
Characteristics of pregnant women		
Age	−0.032 (−0.048–-0.016)	<0.001
Trimesters (0 = first trimester 1 = second trimester 2 = third trimester)	0.162 (0.044–0.280)	0.007
BMI (BMI < 18.5 as reference)		
18.5 ≤ BMI < 25	−0.352 (−0.905–0.200)	0.211
25 ≤ BMI < 30	−0.504 (−1.058–0.050)	0.074
BMI ≥ 30	−0.466 (−1.027–0.095)	0.103
Completed compulsory education (1 = yes)	0.010 (−0.130–0.151)	0.886
First pregnancy (1 = yes)	0.092 (−0.060–0.245)	0.234
Employed (1 = yes)	−0.026 (−0.251–0.200)	0.824
Living with husband (1 = yes)	0.014 (−0.123–0.151)	0.842
Family asset in lowest 25% (1 = yes)	−0.079 (−0.230–0.072)	0.305
Distance to hospital	−0.017 (−0.075–0.042)	0.577
Survey round (0 = survey_Mar2021 1 = survey_Dec2021 2 = survey_Mar2023)	0.048 (−0.027–0.123)	0.209

Survey round is categorical variable indicating the timing of surveys conducted among rural pregnant women. Data were collected during three phases: 0 = March 2021, 1 = December 2021, and 2 = March 2023, with each phase involving different participants. Coefficients are estimated using multiple linear regression, with 95% confidence intervals (CI) reported.

## 4 Discussion

This study provides a comprehensive overview of the health of pregnant women in rural northwestern China by examining three key aspects: nutrition and health knowledge, health practices, and health outcomes. Additionally, it assesses the effectiveness of maternal health services, including received public services and policy awareness. The results highlight that low policy awareness is a major factor contributing to the low rate of folic acid consumption. While received public services and policy awareness are associated with improved nutritional and health knowledge, they do not significantly influence prenatal checkups or health outcomes. Notably, policy awareness is linked to higher rates of picking up free folic acid.

These findings are consistent with previous research showing that maternal health services, particularly received public services and policy awareness, can improve pregnant women’s nutrition and health knowledge (39–41). This suggests that the dissemination of information through health services is effective to some extent. We find that pregnant women’s nutrition and health knowledge may be related to pregnancy order, as first-time mothers appear to be more proactive in learning about maternal and infant health (35, 42), which could explain the association between first pregnancy and higher nutrition and health knowledge. However, our study also finds that the improved knowledge does not necessarily translate into better health behaviors or outcomes, which points to a gap between knowledge acquisition and behavior change.

The failure to translate received public services and policy awareness into improved health practices can be attributed to several factors, including limited economic resources and low educational attainment (43–46). Many rural women face barriers such as inadequate transportation, long distances to healthcare facilities, and high out-of-pocket costs for certain prenatal services, even when subsidies or free care are available. In some cases, traditional family practices take precedence over public health services (47), which may diminish the uptake of services like regular prenatal checkups. These barriers reduce the likelihood of consistent health service utilization and limit the effectiveness of maternal health programs in rural areas.

Although the rate of picking up free folic acid is relatively high, the actual usage among pregnant women remains suboptimal. This is in line with other studies conducted in rural areas (48, 49), where only 44.4% of women took folic acid before and during pregnancy, despite 75.0% having collected the supplements (26). This discrepancy suggests that a large portion of the free folic acid distributed may go unused. Behavioral economics, particularly the sunk cost theory, helps explain this phenomenon. According to the theory (50, 51), individuals are more likely to value and use products they have invested in, even when the cost is irrelevant to future use. Since free folic acid requires no financial investment, pregnant women may feel less motivated to consistently use it, despite its potential health benefits. This lack of cost creates a lower perceived value, which can lead to greater waste and lower adherence to recommended supplementation.

Our findings also suggest that policy awareness is critical in boosting both the collection and usage of free folic acid supplements among pregnant women. In rural areas, access to and consumption of folic acid is largely dependent on awareness of relevant health policies (52). This highlights the need for more effective dissemination strategies to ensure that women not only pick up the supplements but also use them appropriately. Therefore, enhancing policy dissemination and awareness campaigns is essential for increasing folic acid consumption and ultimately improving maternal health outcomes (53).

Despite the positive impact of policy awareness on health knowledge and supplement uptake, our study finds that neither received public services nor policy awareness significantly improves the overall health status of pregnant women. Research has shown that although most rural women in China are aware of the importance of prenatal care and folic acid supplementation, there remains a significant gap between awareness, practices, and actual health improvements (52, 54, 55). This gap can be better understood through the lens of perceived barriers (56). Perceived barriers refer to the tangible difficulties that hinder individuals from taking action, even when they understand the importance of health behaviors. Previous studies have identified that limited healthcare facilities, high costs, and transportation challenges often present significant obstacles for pregnant women seeking prenatal checkups or obtaining folic acid supplements (57–59). These practical factors may substantially diminish the effectiveness of public health services, as individuals may refrain from acting on their health knowledge if they perceive the effort to overcome these barriers as too burdensome (60).

Moreover, social norms play a critical role in shaping the health behaviors of pregnant women. In rural areas, traditional beliefs and customs can profoundly influence women’s decisions, even when they are well informed about the benefits of folic acid supplementation. Women may face resistance from family or community members, particularly when these individuals hold divergent health views or lack relevant knowledge (61). This phenomenon aligns with existing research, which demonstrates that health decisions are influenced not only by individual knowledge but also by social behaviors and collective expectations (62–64).

Bridging the gap between health knowledge and behaviors among pregnant women is an area deserving greater policy attention. This can be achieved by improving the accessibility of healthcare services and implementing family-centered health education programs, thereby reducing perceived barriers and reshaping social norms. These measures are vital to enhancing the effectiveness of maternal and child public health services in rural areas.

This study has several limitations. First, our research subjects are from rural areas in Shaanxi, China, so the findings may not be generalizable to other regions. Second, due to data limitations, we categorized received public services and policy awareness as binary variables based solely on participant responses. This simplified approach prevents us from capturing the quality of maternal health services, which may have impacted the accuracy and depth of our results to some extent. Future research should focus on incorporating service quality indicators to provide a more nuanced understanding of the effectiveness of maternal health programs in rural settings. Third, due to data limitations, we were unable to control for potential confounding variables such as access to healthcare beyond public services, cultural and social factors, and the level of family support, which might influence the study results. Future research should focus on more comprehensive data collection, incorporating qualitative research methods, and adopting stratified analyses or study designs with stronger causal inference to explore the role of these variables and further elucidate their mechanisms of influence. Fourth, as the data on pregnant women’s health practices and outcomes were collected retrospectively through self-reports, there is a possibility of recall bias, which may have affected the accuracy of the measurements.

## 5 Conclusion

In summary, our findings indicated that despite a high rate of picking up free folic acid, the actual taking of folic acid among rural pregnant women was unsatisfactory. Both received public services and policy awareness significantly enhanced the nutritional health knowledge of pregnant women. Additionally, policy awareness promoted picking up free folic acid and its taking. However, neither received public services nor policy awareness had a significant impact on prenatal checkups or the health outcomes of pregnant women.

## Data Availability

The datasets used and/or analyzed during the current study are available from the corresponding author on reasonable request.
